# The intellectual structure and emerging trends on nanotechnology in inflammatory bowel disease: A bibliometric analysis from 2005 to 2024

**DOI:** 10.1097/MD.0000000000049947

**Published:** 2026-07-24

**Authors:** Lei Liang, Dexin Wang, Xiubi Zhang, Heng Yang, Xiaohe Zhang, Lan Li, Gang Tian, Chaochi Yue, Weiliang Du

**Affiliations:** a College of Integrative Chinese and Western Medicine, Southwest Medical University, Luzhou, Sichuan, China; b Affiliated Hospital of North Sichuan Medical College, Nanchong, Sichuan, China; c Department of Laboratory Medicine, Sichuan Province Engineering Technology Research Center of Molecular Diagnosis of Clinical Diseases, Molecular Diagnosis of Clinitional Chinese Medicine, The Affiliated Hospital of Southwest Medical University, Luzhou, Sichuan, China; d Department of Traditional Chinese Medicine, The Affiliated Hospital of Southwest Medical University, Luzhou, Sichuan, China.

**Keywords:** bibliometrics, drug delivery system, inflammatory bowel disease, nanoparticles, nanotechnology

## Abstract

**Background:**

As a chronic inflammatory disease of the intestine, inflammatory bowel disease (IBD) is challenged by existing treatment methods, such as poor drug targeting, low bioavailability, and systemic toxicity. In recent years, nanotechnology has provided a new strategy for the treatment and diagnosis of IBD due to its advantages of precise delivery, controllable release and multifunctional integration.

**Methods:**

We searched the Web of Science Core Collection database for relevant literature about nanotechnology and IBD published from 2005 to 2024. We used SciExplorer, VOSviewer, and Citespace to analyze countries, institutions, authors, keywords, highly cited references, and co-cited references to discuss research hotspots and trends in this field.

**Results:**

The research analysis included a total of 959 pieces of literature, with China and the USA leading in the number of papers published and academic influence. The Georgia State University had the most papers posted. Merlin Didier and Xiao Bo were the scholars who published the most. The research hotspots mainly focus on nanodrug delivery systems, targeted therapy and inflammation regulation, while exosomes, macrophage polarization, and intestinal microbiota regulation are becoming new research frontiers. In the future, intelligent responsive nanomaterials, multifunctional nanoplatforms, and personalized nanotechnology will become important development directions.

**Conclusion:**

A new avenue for the accurate diagnosis and treatment of IBD has been unlocked by nanotechnology, but its clinical translation needs to break through the bottlenecks of biocompatibility, large-scale preparation and interdisciplinary collaboration. In the future, we should focus on the development of “intelligent responsive nanosystems,” deepen the research on the interaction mechanism of “nano-microbe-host,” and promote the establishment of a personalized treatment system.

## 1. Introduction

Inflammatory bowel disease (IBD) is a complex group of diseases characterized by chronic, recurrent intestinal inflammation, mainly including Crohn disease (CD) and ulcerative colitis.^[1]^ The symptoms of IBD include abdominal pain, diarrhea, blood in the stool, and weight loss, etc, which are prolonged and may cause serious complications such as intestinal fibrosis and cancer.^[2]^ The prevalence of IBD is on the rise annually, particularly in industrialized countries.^[3]^ The exact origins of IBD are still unclear, but it is commonly thought to arise from a blend of genetic susceptibility, intestinal dysbiosis, immune system irregularities, and external factors.^[4–6]^ At present, immunosuppressants such as glucocorticoids and biologics are the main treatment methods for IBD, but long-term use can easily lead to increased risk of infection, drug resistance and systemic side effects, and some patients have insufficient response to existing therapies.^[7–9]^ Therefore, there is an urgent need to develop novel therapeutic strategies that are more targeted, safe, and highly effective.

Nanotechnology is an interdisciplinary discipline that combines nanotechnology and medicine, aiming to achieve accurate diagnosis, treatment, prevention and health monitoring of diseases through the design and application of nanoscale materials, devices and systems, usually at the scale of 1 to 100 nm.^[10,11]^ In recent years, the rapid advancement of nanotechnology has provided a revolutionary idea for the diagnosis and treatment of IBD. By designing nanoscale functional materials such as liposomes, polymer nanoparticles, metal-organic frameworks, etc, researchers can achieve precise drug delivery, responsive release of inflammatory microenvironment, and integration of multimodal diagnosis and treatment.^[12]^ For example, based on the enhanced epithelial permeability of intestinal inflammation sites, nanocarriers can be enriched in the lesion area through passive targeting (enhanced permeability and retention effect).^[13]^ Targeting molecules through surface modifications can further achieve active targeting, significantly increasing local drug concentrations and reducing systemic exposure.^[14]^ In addition, nanotechnology has also been used to modulate the intestinal immune microenvironment, such as the delivery of anti-inflammatory cytokines, silencing pro-inflammatory genes, and regulating intestinal microbiota balance.^[15]^ In the field of diagnostics, nanoprobes can be used to noninvasively monitor intestinal inflammatory activity and molecular markers by combining optics, magnetic resonance imaging, or biosensor technology, helping to facilitate the early diagnosis and individualized treatment of IBD.^[16,17]^ However, the application of nanotechnology in IBD still faces challenges. The in vivo metabolism mechanism, long-term biosafety and large-scale production process of nanomaterials still need to be further explored.^[18]^ The complex physicochemical environment of the intestinal tract, such as mucus layer barrier and microbiota metabolism interference may affect the stability and targeting efficiency of nanocarriers.^[19]^ Future research needs to combine multi-omics analysis, organoid models, and artificial intelligence design to promote the clinical transformation of nano-diagnosis and treatment systems and provide more accurate treatment plan for IBD patients.^[20,21]^

As a method of quantitative analysis of scientific literature, bibliometrics can reveal the development context, research hotspots and future trends of a certain research field.^[22]^ At present, although there have been a large number of studies on the application of nanotechnology in IBD, no studies have systematically analyzed this field from the perspective of bibliometrics at present. Therefore, this study aims to comprehensively sort out the application status of nanotechnology in IBD research through bibliometric methods, identify key research strengths, analyze research hotspots and frontiers, and provide reference and enlightenment for the future development of this field.

## 2. Methods

### 2.1. Search strategy

**Figure 1.**
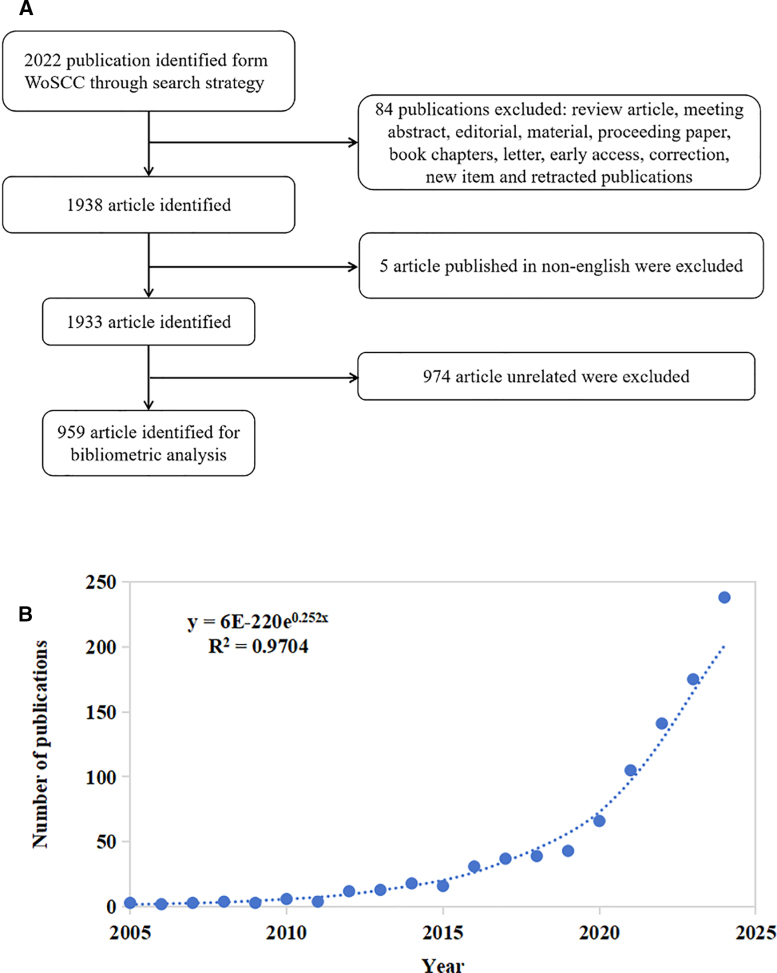
(A) Literature screening flowchart. (B) Analysis of the annual publication index. WOSCC = Web of Science Core Collection.

The literature for this study is sourced from “English” papers published on the Web of Science Core Collection (WOSCC) between 2005 and 2024. The required types of literature are “Article” or “Review article.” We conducted searches using IBD and nanotechnology subject words. The specific subject words and retrieval formula are as follows: TS= ([“Nanotechnol*” OR “Nanoparticle*” OR “Nanomaterial*” OR “Nanocarrier*” OR “Liposome*” OR “Dendrimer*” OR “Exosome*” OR “Micelle*” OR “Quantum dot*”] AND [“Inflammatory Bowel Disease” OR “IBD” OR “Crohn*Disease” OR “Ulcerative Colitis”]). The time limit is from January 1, 2005 to December 31, 2024. Ultimately, the data is exported in “plain text” and “tab character” formats, including “full records and citations.” Literature screening was conducted independently by 3 researchers, and each article was manually reviewed to ensure that only relevant publications were included. Articles were excluded if they fell under categories such as conference proceedings, book chapters, retracted articles, editorials, errata, or letters. In response to the differences of opinion of the reviewers, we have established a standardized resolution process. First, the reviewers discuss based on inclusion/exclusion criteria to seek consensus. If no agreement is reached, it is submitted to senior researchers for independent review. In the end, the research team will make a decision on whether to include it based on the opinions of the third party, which effectively ensures the objectivity of the screening process and the credibility of the results. We performed automated and manual deduplication via Excel sheets and conducted a rapid review of randomly selected samples against predefined inclusion/exclusion criteria to assess the effectiveness of the search strategy. Ultimately, 959 articles were deemed suitable for bibliometric analysis and key literature content analysis. The flowchart illustrating the data collection and filtering process can be seen in Figure 1A.

### 2.2. Software for analysis

This study used SciExplorer online site, Citespace (version 6.3.1). VOSviewer (version1.6.19) and 114 Scimago Graphica (version1.0.42) for analysis. VOSviewer has facilitated the creation of a collaborative network involving authors, countries, and institutions. After cleaning the data by merging synonyms, keywords were clustered and analyzed for co-occurrence using CiteSpace. Additionally, data from VOSviewer were imported into Scimago Graphica for analyzing the strength of national cooperation.

VOSviewer is a free and open-source literature visualization tool developed by Leiden University in the Netherlands, based on the idea of Visual Mapping of Science, which can help researchers quickly identify research hotspots, analyze the cooperation patterns between authors and institutions, reveal the knowledge relationship between literature, and track the development trend of the research field through co-occurrence network analysis, cooperative network analysis, citation analysis, and other functions.^[23]^ It supports importing data from databases such as Web of Science, Scopus, CNKI, etc, and generates clear analysis maps through data cleaning, multiple visualization views (such as network view, label view, and density view), and finally exports high-quality images for reports or presentations. Due to its powerful functions and ease of use, VOSviewer has become an important tool for researchers to analyze literature data and build knowledge graphs, and is widely used in academic research and bibliometric analysis.

CiteSpace is a scientific literature analysis tool developed by Professor Chaomei Chen, which is used to analyze literature data through visual means to help researchers quickly identify hot spots, cutting-edge topics, key documents and cooperation networks in the research field.^[24]^ It supports the import of data from databases such as Web of Science, Scopus, CNKI, etc, and generates a scientific knowledge graph through high-frequency keyword analysis, co-citation analysis, citation prominence detection and other functions, revealing the core themes, hot spot evolution, and frontier directions of the research field.^[25]^ CiteSpace is widely used in academic research and technical analysis, and its powerful visualization capabilities make it an important tool for researchers to sort through knowledge and explore academic frontiers.

SciExplorer is an open scientometric data visualization and analysis platform developed by the Library and Information Center of the Chinese Academy of Sciences, which aims to help users quickly analyze and visualize scientific and technological literature data through scientometric principles and data visualization technology. The platform consists of 3 subsystems: LDGAS data governance and statistical analysis system, KMVS knowledge matrix visualization system, and MSSS scientific structure diagram overlay analysis system, which are used for data cleaning and statistical analysis, knowledge matrix construction and visualization, and scientific field structure diagram generation and overlay analysis, respectively. SciExplorer helps users grasp the research direction from a macro perspective through intuitive visualization methods, reveals scientific and technological information in literature data, and supports scientific research situational awareness and decision-making.

## 3. Results

### 3.1. Publication trends

This study comprehensively examined a total of 959 articles focusing on the intersection of IBD and nanotechnology. As depicted in Figure 1B, the overall trend in the number of publications related to IBD and nanotechnology has shown a consistent upward trajectory from 2005 to 2024, highlighting the growing interest and advancements in this field over the past 2 decades. Since 2016, there has been a slow increase in the number of published papers, but it has rapidly escalated since 2019, with 238 articles in 2024, demonstrating a significant rise in researchers’ focus on this field since 2019. To explore the relationship between the publication year and the growth trend of articles, the annual cumulative number of publications was meticulously evaluated using an exponential growth function. Figure 1B demonstrates a strong correlation trend (*R*^2^ = 0.9704), indicating significant progress in IBD and nanotechnology research. This suggests a rising interest in the field over the last 2 decades.

### 3.2. Analysis of journals

**Figure 2.**
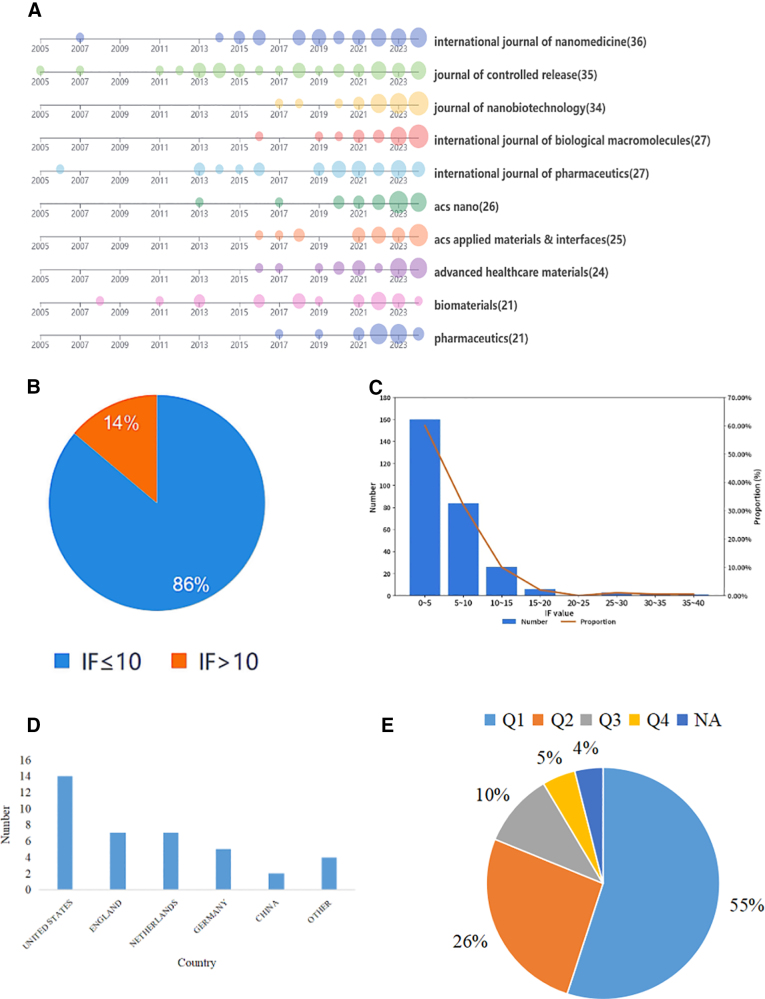
(A) Journal publication trends. The larger the circle, the more posts are posted. (B) The proportion of journals with an IF >10. (C) Journal IF analysis. (D) Proportion of journals with high impact factor published in countries. (E) Proportion of JCR partitions.

Papers on nanotechnology for IBD treatment are predominantly found in materials science and medical journals. The *Journal of Nanobiotechnology* and *International Journal of Biological Macromolecules* have experienced the fastest increase in published articles in this field over the past 5 years (Fig. 2A). Figure 2A and Table 1 show that the highest number of publications on the subject were published in the *International Journal of Nanomedicine* (36 publications), followed by the *Journal of Controlled Release* (35 publications), and the *Journal of Nanobiotechnology* (34 publications). All of the top 10 journals in terms of publication volume were in the Q1 division (2024 JIF quartile), and each of these top-ranked journals had an impact factor (IF) exceeding 5. This suggests that the use of nanotechnology in IBD is well-regarded by high-quality journals. On the flip side, the top 5 journals that cited articles under study were *Journal of Controlled Release*, *Biomaterials*, *International Journal of Pharmaceutics*, *International Journal of nanotechnology*, and *Acs Nano*. The top 10 journals featured several leading publications in the field, including *Acs Nano*, *Biomaterials*, *Journal of Nanobiotechnology*, and *Journal of Controlled Release*. This suggests that nanotechnology is well-grounded in theory for addressing IBD.

**Table 1:** Information of the first 20 journals publications.

Rank	Label	Count	IF (2024)	JCR	Country	Citation
1	*International Journal of Nanomedicine*	36	6.6	Q1	New Zealand	979
2	*Journal of Controlled Release*	35	10.5	Q1	Netherlands	2310
3	*Journal of Nanobiotechnology*	34	10.6	Q1	England	783
4	*International Journal of Biological Macromolecules*	27	7.7	Q1	Netherlands	381
4	*International Journal of Pharmaceutics*	27	5.3	Q1	Netherlands	1194
6	*Acs Nano*	26	15.8	Q1	United States	909
7	*Acs Applied Materials & Interfaces*	25	8.3	Q1	United States	727
8	*Advanced Healthcare Materials*	24	10.1	Q1	Germany	462
9	*Biomaterials*	21	12.8	Q1	Netherlands	2296
9	*Pharmaceutics*	21	4.9	Q1	Switzerland	293
11	*Journal of Drug Delivery Science and Technology*	20	4.5	Q1	France	285
12	*Carbohydrate Polymers*	16	10.7	Q1	England	780
12	*Chemical Engineering Journal*	16	13.3	Q1	Switzerland	231
14	*Journal of Materials Chemistry B*	15	6.1	Q1	England	248
15	*Biomaterials Science*	14	5.8	Q1	England	229
16	*European Journal of Pharmaceutics and Biopharmaceutics*	13	4.4	Q1	Netherlands	430
17	*Acta Biomaterialia*	12	9.4	Q1	England	246
17	*International Journal of Molecular Sciences*	12	4.9	Q1	Switzerland	252
19	*Science Advances*	11	11.7	Q1	United States	882
20	*Drug Delivery*	10	6.5	Q1	United States	208
20	*Theranostics*	10	12.4	Q1	Australia	811

IF = impact factor.

Through the statistical analysis of the IF of journals, there are 39 journals with an impact factor >10, accounting for 14% of the total journals, and 281 articles came from these journals, accounting for 29.30% of the total number of articles published in the past 20 years (Fig. 2B). The proportion of journals with IF between 0 and 5 is the largest, and the higher the IF, the fewer the number of journals (Fig. 2C). It is particularly noteworthy that the United States is the country with the highest number of high-IF journals (N = 14.36%), followed by the Netherlands (N = 7.18%), and the England (N = 7.18%; Fig. 2D). Although China has published the largest number of publications, there are few high-quality journals on the application of nanotechnology in the field of IBD. Journals in the JCR subdivision of Zone 1 accounted for 54.96% of the total journals (Fig. 2E). This indicates that there are many high-quality literatures in this field, and the relevant research is of high quality, which has been widely recognized by top journals.

### 3.3. Analysis of countries

**Figure 3.**
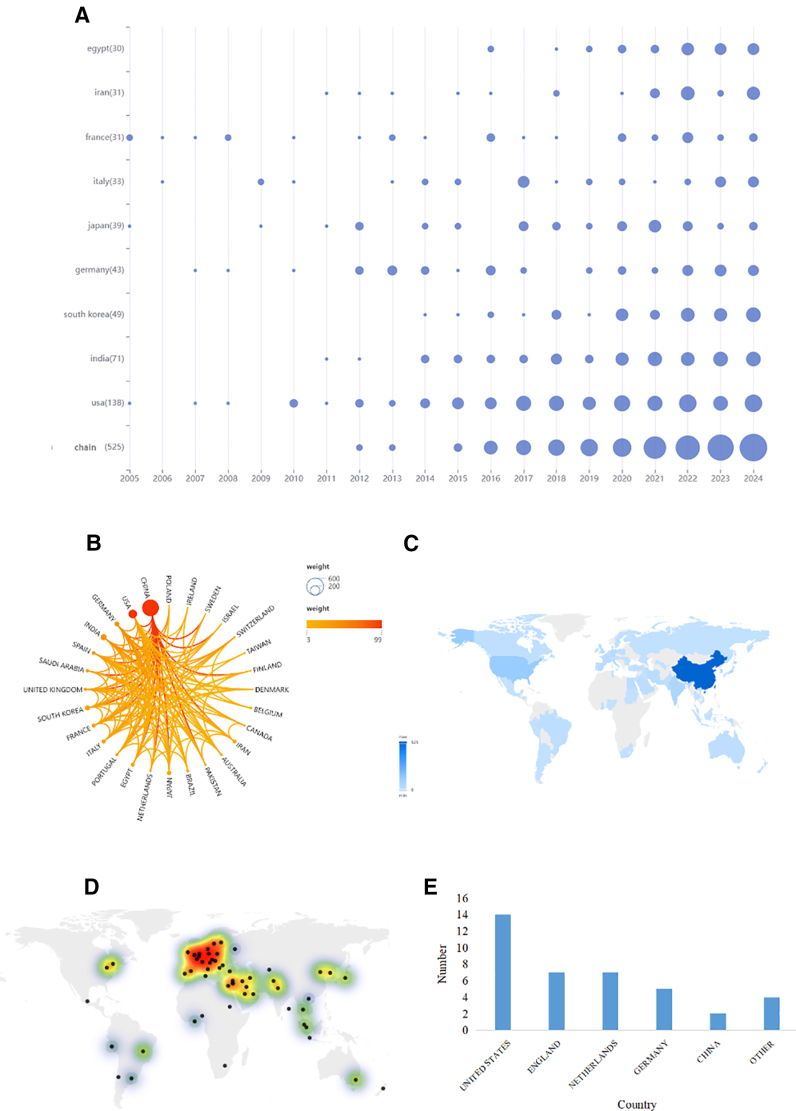
(A) Annual publication volume of the top 10 countries. (B) National cooperation network map. (C) National Geographic visualization. (D) National density map of publication. (E) Number of journals in different publishing countries.

Investigating the number of research publications on IBD and nanotechnology, along with collaborative networks, was the main objective of this study. Figure 3A shows that China ranks first (525 publications, 54.74%), followed by the United States (136 publications, 14.18%), and India (54 publications, 7.40%). The remaining countries and regions each had fewer than 50 publications. A country collaboration network analysis was conducted with 28 countries meeting a minimum publication threshold of 5. The China rose as the key player in this domain, establishing close collaborations with United States, India, Germany and so on. Its total link strength of 99 was considerably higher than that of other countries (Fig. 3B). It can also be seen from the national geographic visualization that the main research country of IBD is China, United States, and developed countries of Europe, and the China as the center (Fig. 3C). With the development of industrialization in China and the change of dietary structure, the incidence of IBD has also increased. This may be one of the reasons for the increase in the number of research and publications in this field in China. In Figure 3D, the redder the color represents the greater the number of countries publishing in the field of IBD and nanotechnology, and in combination with Figure 3E, the largest number of publishing countries are in Europe, which shows that Europe is more concerned about the development of this field than other continents.

### 3.4. Analysis of institutional

**Figure 4.**
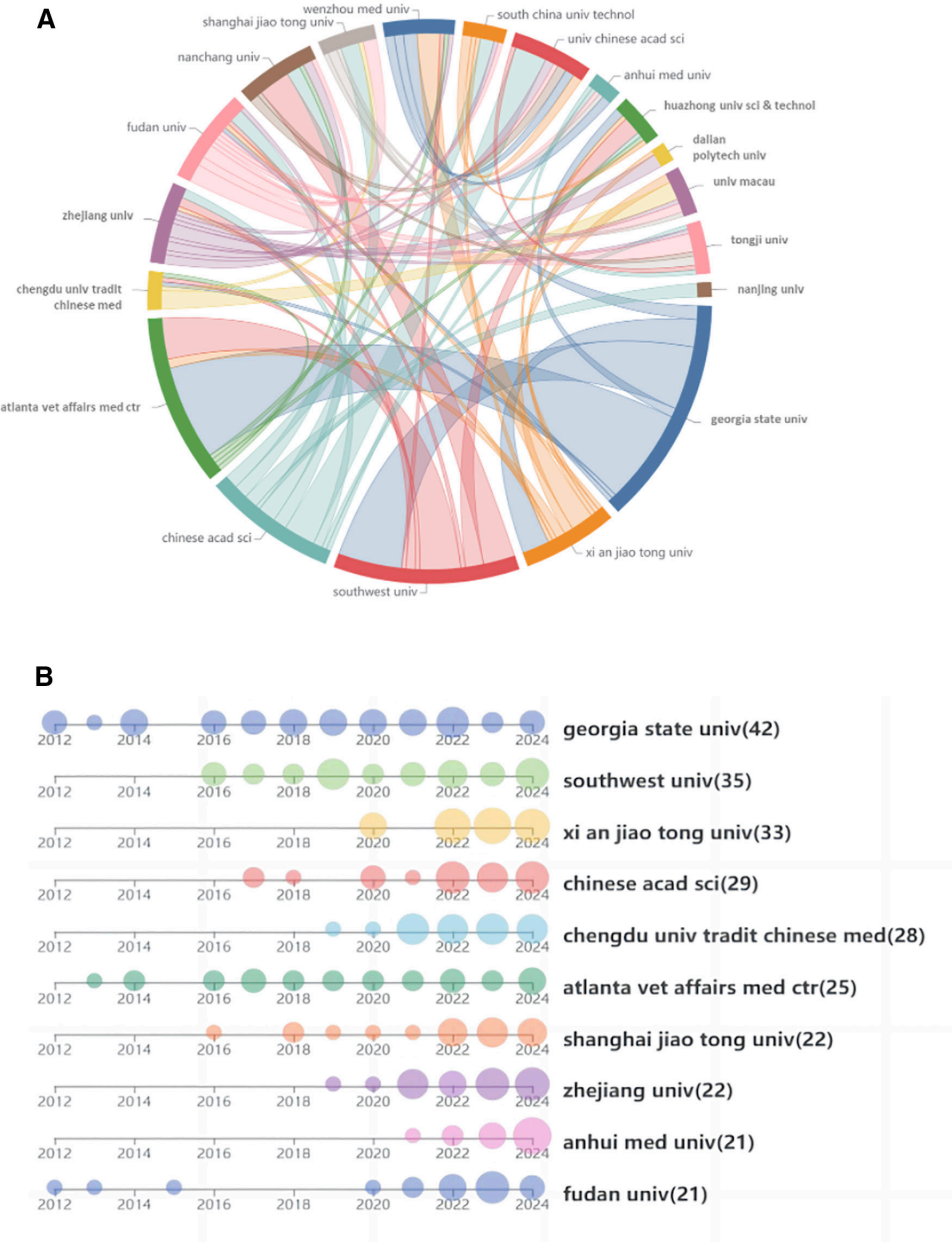
(A) Institutional cooperation string chart. (B) Institutional article issuance trend.

In Figure 4A, the Georgia State University (42 publications, 4.37%), Xi’an Jiao Tong University (38 publications, 3.96%), Southwest University (35 publications, 3.64%), University of Chinese Academy of Sciences (29 publications, 3.02%), and Chengdu University Traditional Chinese Medical (28 publications, 2.91%) are the top 5 institutions in terms of the number of publications. Of the top 20 institutions, 16 are from China, and these institutions work closely together. The most closely cooperating institutions are Georgia State University and Atlanta Department of Veterans Affairs Medical Center, and we found that there is close cooperation between Chinese universities. In Figure 4B, we can study that Xi’an Jiao Tong University and Chinese Academy of Science have published the most articles in the past 5 years. Georgia State University has published articles almost every year since 2012, demonstrating the institution’s ongoing interest in nanotechnology and IBD, as well as a better theoretical and experimental basis than other institutions.

### 3.5. Analysis of authors

**Figure 5.**
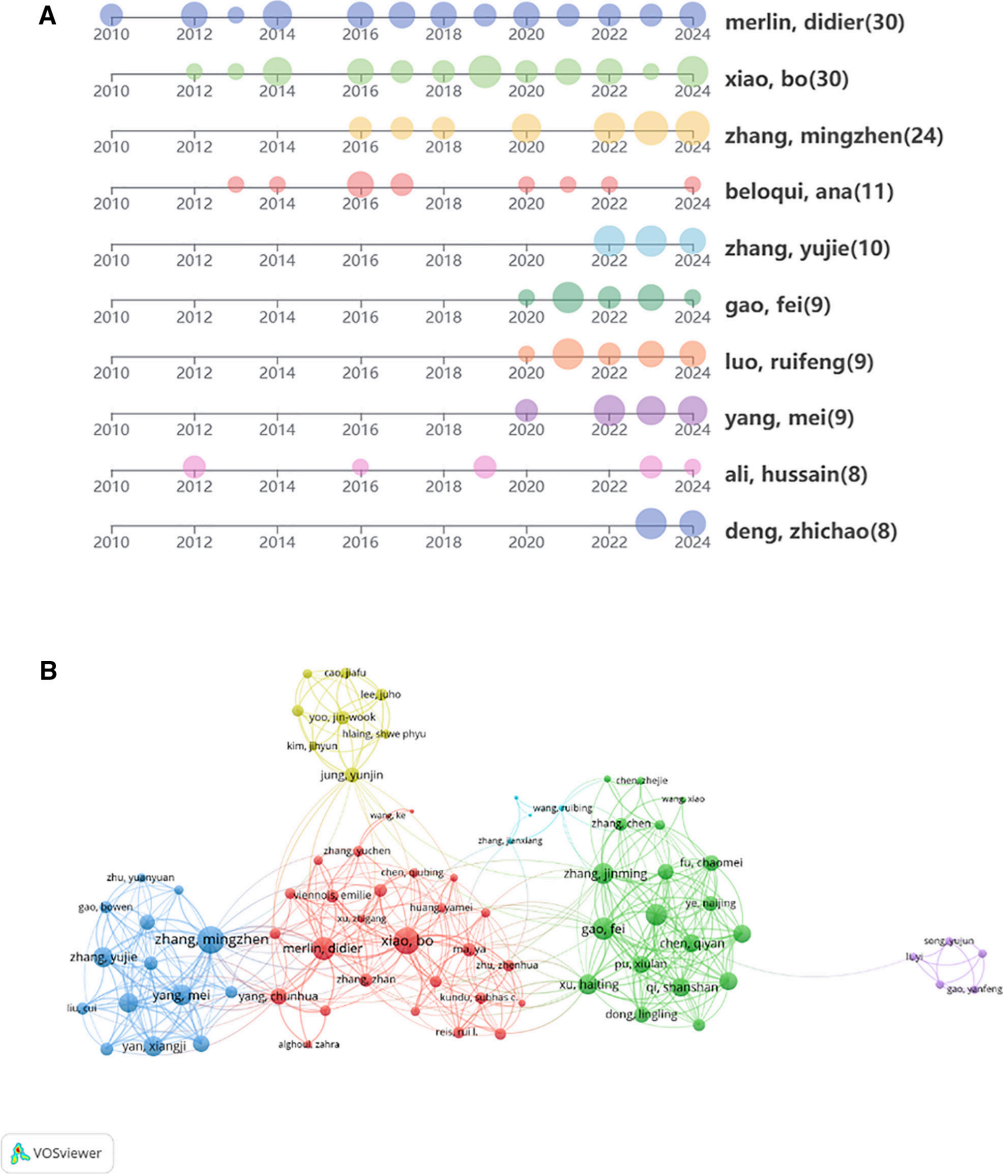
(A) the trend of authors’ articles. (B) Author clustering.

Figure 5A illustrates the number of publications by the top of 10 authors, and Figure 5B illustrates the cooperation network among authors. Merlin, D and Xiao, B (33 publications, 3.44%), Zhang, MZ (30 publications, 3.12%), Ma Y (14 publications, 2.82%), and Zhang Y (23 publications, 2.40%) are the top 5 authors. The size and color of the nodes in Figure 5B can represent centrality. The larger the node and the thicker the line, which means that the centrality is higher, indicating that the cooperation between the authors is stronger. The collaboration between the top 5 authors is lack, almost all have their own circle of cooperation.

Figure 5A systematically presents the publication output of the top 10 most productive authors in the field. Notably, Merlin D and Xiao B lead with 33 publications (3.44% of total output), followed by Zhang MZ (30 publications, 3.12%), Ma Y (14 publications, 2.82%), and Zhang Y (23 publications, 2.40%). The visualization highlights their distinct academic productivity through proportional bar lengths and percentage annotations. Figure 5B further reveals collaborative patterns through a network analysis where node size and chromatic intensity correspond to author centrality. Larger nodes with thicker connecting lines indicate higher centrality values, reflecting stronger collaborative relationships within the network. Notably, while the top 5 authors demonstrate baseline collaborative engagement, analysis reveals relatively independent cooperative clusters.

### 3.6. Correlation analysis of key papers

The rising citation frequency of publications serves as a quantitative indicator of their scientific credibility and methodological robustness, reflecting their growing acceptance as standardized references within the field. A thematic analysis of highly cited works offers a strategic lens to map the current intellectual landscape of nanotechnology applications in IBD, while simultaneously identifying emerging trends and knowledge gaps that may guide future investigations. Table 2 illustrates the top 10 highly cited references. Most of the pivotal literature focuses on nanocarrier delivery and targeted therapies. The top cited article is named “Orally delivered thioketal-nanoparticles loaded with TNFα-siRNA target inflammation and inhibit gene expression in the intestines.” Niren M et al^[26]^ developed sulfate nanoparticle carriers that inhibit gene expression in diseased tissues by localizing silencing pro-inflammatory genes to sites of intestinal inflammation, and the polymer selectively degrades reactive oxygen species (ROS). The article “Combination Therapy for Ulcerative Colitis: Orally Targeted Nanoparticles Prevent Mucosal Damage and Relieve Inflammation” describes the team’s development of a hyaluronic acid-functionalized polymer nanoparticle for simultaneous delivery of siRNA and a chemical drug to key cells associated with ulcerative colitis treatment.^[27]^ At the same time, the study found that the combination of siCD98 and curcumin can prevent mucosal damage, inhibit the overexpression of CD98 and TNF-α genes, and has better efficacy than a single drug preparation. James JM et al.^[7]^ Reported that hyaluronic acid-bilirubin nanodrugs can modulate the intestinal microbiota and increase the abundance and diversity of intestinal microbiota, such as the increased abundance of *Akkermansia muciniphila* and *Clostridium* XIVα. In addition to this, it also has the effect of regulating innate immunity and repairing the intestinal barrier. Didier M et al^[28]^ found that oral GDNPs 2 is a novel delivery mechanism in improving the prevention and treatment of IBD and overcomes the potential toxicity and production scale limitations common to synthetic nanoparticles. In the study, it was found that GDNPs 2 increased the survival and proliferation of intestinal epithelial cells, decreased pro-inflammatory cytokines (TNF-α, IL-6, and IL-1β), and increased anti-inflammatory cytokines IL-10 and IL-22, thereby achieving efficacy. Hui W et al^[29]^ developed an integrated cascade of Pt@PCN222-Mn nanozymes, which revealed that this nanozyme has a good ROS clearance ability in the treatment of inflammation in vivo through in vitro and in vitro experiments, and verified its efficacy on IBD, which provides a new prospect for the future application of integrated nanozymes with multifunctional active sites.

**Table 2:** The top 10 key article information.

Rank	Title	Author	Year	Citation	DOI
1	Orally delivered thioketal nanoparticles loaded with TNF-α-siRNA target inflammation and inhibit gene expression in the intestines.	Wilson DS, Dalmasso G, Wang LX, et al	2010	581	10.1038/nmat2859
2	Edible ginger-derived nanoparticles: A novel therapeutic approach for the prevention and treatment of inflammatory bowel disease and colitis-associated cancer.	Zhang MZ, Viennois E, Prasad M, et al	2016	569	10.1016/j.biomaterials.2016.06.018
3	Hyaluronic acid-bilirubin nanomedicine for targeted modulation of dysregulated intestinal barrier, microbiome and immune responses in colitis.	Lee Y, Sugihara K, Gillilland MG, et al	2020	467	10.1038/s41563-019-0462-9
4	Advances in oral nano-delivery systems for colon targeted drug delivery in inflammatory bowel disease: selective targeting to diseased versus healthy tissue.	Hua S, Marks E, Schneider JJ, et al	2015	403	10.1016/j.nano.2015.02.018
5	Integrated cascade nanozyme catalyzes in vivo ROS scavenging for anti-inflammatory therapy.	Liu YF, Cheng Y, Zhang H, et al	2020	372	10.1126/sciadv. abb2695
6	An inflammation-targeting hydrogel for local drug delivery in inflammatory bowel disease.	Zhang SF, Ermann J, Succi MD, et al	2015	340	10.1126/scitranslmed.aaa5657
7	Effect of surface chemistry on nanoparticle interaction with gastrointestinal mucus and distribution in the gastrointestinal tract following oral and rectal administration in the mouse.	Maisel, K; Ensign, L; Reddy, M; et al	2015	258	10.1016/j.jconrel.2014.10.026
8	Dextran sodium sulfate (DSS) induces colitis in mice by forming nano-lipocomplexes with medium-chain-length fatty acids in the colon.	Laroui H, Ingersoll SA, Liu HC, et al	2012	248	10.1371/Journal.pone.0032084
9	Cell specific delivery of modified mRNA expressing therapeutic proteins to leukocytes.	Veiga N, Goldsmith M, Granot Y, et al	2018	230	10.1038/s41467-018-06936-1
10	An Orally Administered CeO 2 @Montmorillonite Nanozyme Targets Inflammation for Inflammatory Bowel Disease Therapy	Zhao S, Li, YX, Liu QY, et al	2020	223	10.1002/adfm.202004692

### 3.7. Analysis of keywords

**Figure 6.**
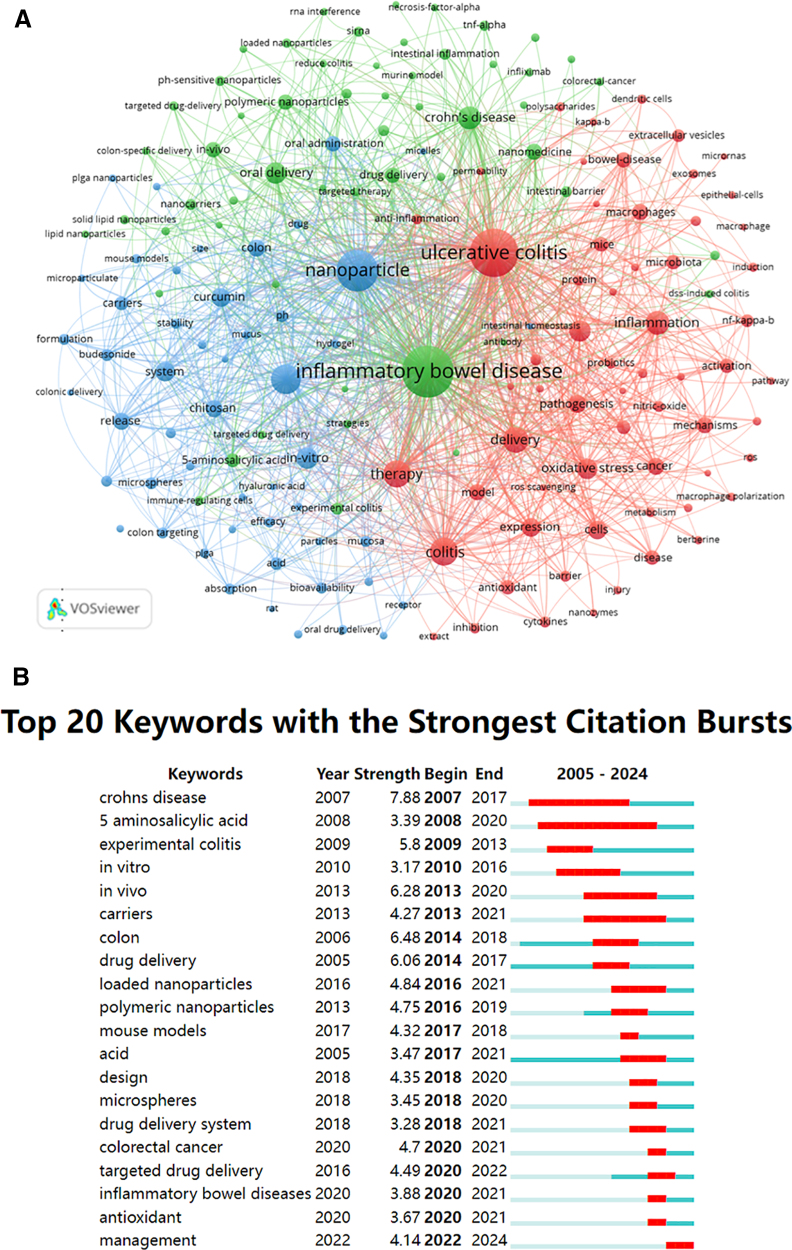
(A) Keyword clustering. (B) Top 20 keywords with the strongest citation bursts. Keywords bursts are marked by the red bars, which indicate their start, end, and duration.

In VOSviewer, the classification of keyword clusters reflects the topic division, hotspots and trends, interdisciplinary and integration, domain structure distribution, and topic evolution in the research field. Figure 6A visualizes keyword co-occurrence through a network analysis, delineating emerging research frontiers of nanotechnology in IBD. The size of the nodes in the figure corresponds to their frequency of keyword occurrence. Table 3 presents the most frequently occurring keywords in the co-occurrence analysis: inflammation bowel disease, nanoparticle, drug delivery, colitis, therapy, and delivery. Keyword burst signifies an explosive increase in research over time, indicating future development trends. Figure 6B illustrates the top 20 keywords with the strongest burst intensity in this study. The red line in the figure represents the duration of the keyword burst. The 5 most powerful keywords include in vivo, IBD, drug delivery, nanoparticle, and targeted therapy. The explosion of keywords signifies a sharp surge in academic attention to a certain field in a given period. Keyword outbreaks in the last 2 years include IBD, antioxidant, targeted drug delivery, and management. Through the analysis of the visualization results of keywords, it can be seen that the current application of nanotechnology in the field of IBD is mainly focused on the study of nanomaterials for the treatment of IBD through antioxidant and targeted delivery of drugs.

**Table 3:** Frequency of keywords occurrence.

Rank	Keyword	Occurrences
1	Inflammatory bowel disease	548
2	Ulcerative colitis	449
3	Nanoparticle	329
4	Drug-delivery	168
5	Colitis	148
6	Therapy	140
7	Delivery	127
8	Inflammation	112
9	Crohn disease	102
10	Gut microbiota	88

## 4. Discussion

### 4.1. General situation analysis

In the early 21st century, there was a gradual emergence of research on applying nanotechnology in IBD therapy. Despite this, the idea failed to attract significant attention, with an average annual publication rate of <10. Nevertheless, 2019 emerged as a pivotal turning point in the field of nanotechnology for IBD therapy. As researchers increasingly recognized the transformative potential of nanotechnology in enhancing IBD treatment efficacy, this area rapidly gained prominence and became a focal point for further exploration. The volume of published papers serves as a key metric to gauge the emergence and popularity of a research field. 2024 marked a peak with a record 238 publications, reflecting the study of this field sustained growth. The annual growth rate of publications from 2004 to 287 2024 was a remarkable 19.47%, underscoring the field’s dynamic expansion. We anticipate that the number of articles on nanotechnology in IBD treatment will continue to rise in the coming years. Among the top 5 countries with the highest publication counts were China, the United States, India, Germany, and South Korea. China led the way with an impressive 525 articles, significantly outpacing other nations and demonstrating a robust citation rate. The United States followed closely, with a substantial number of publications. Notably, the average citation per article in the United States surpassed that of China, indicating that its research findings were highly attention within the academic community. The majority of nanotechnology for the treatment of IBD research has been published in high-impact journals, including the *International Journal of Nanotechnology* (2024 IF = 6.6), *Journal of Controlled Release* (2024 IF = 10.5), *Journal of Nanobiotechnology* (2024 IF = 10.6), *International Journal of Pharmaceutics* (2024 IF = 5.3), and *International Journal of Biological Macromolecules* (2024 IF = 7.7). Moreover, the top 20 journals with the highest publication volumes in this field were all ranked in the Q1 quartile (2024 JIF quartile), highlighting the high quality and broad recognition of these studies by leading journals. Keywords analysis revealed that nanoparticles, delivery, gut microbiota, macrophages, oxidative stress, nanotechnology, drug-delivery, and polymeric nanoparticles were steering the research filed. A significant correlation existed between these keywords. It can be seen that the key to nanotechnology therapy of IBD lies in drug delivery, targeted drug delivery, gut microbiota, oxidative stress, antioxidant, anti-inflammatory, etc. In addition, there are also differences in research priorities in different years. Before 2019, the keyword outbreak was mainly reflected in 5-aminosalicylic acid, in vivo, loaded nanoparticles, acid, polymeric nanoparticles, etc, targeted drug delivery from 2020, and antioxidant is the main keyword, which can be seen in the past 4 years. Research in the field mainly focuses on targeted drug delivery and scavenging of ROS.

### 4.2. Drug delivery system

One of the core advantages of nanotechnology in the treatment of IBD is the optimization of drug delivery efficiency. Traditional oral drugs are susceptible to degradation or nonspecific distribution in the gastrointestinal tract, resulting in low bioavailability and significant systemic side effects.^[30,31]^ Nanocarriers, such as liposomes, polymer nanoparticles, mesoporous silica, and so on can prolong the intestinal retention time by protecting the drug from gastric acid and enzymatic hydrolysis, and maintain the local effective concentration through the sustained-release mechanism to achieve controlled drug release.^[32]^ For example, pH-responsive nanoparticles release drugs in areas of inflammation in the gut, while enzyme-sensitive carriers enable precise drug release in the context of enzyme overexpression at the site of inflammation.^[33,34]^ In addition, nanoparticles can increase the penetration of drugs in biological barriers, so that drugs can reach the lesion area more efficiently.^[35]^ However, the large-scale production, long-term safety and transmucosal permeability efficiency of nanocarriers still need to be further optimized.

### 4.3. Targeted therapy

The active targeting strategy of nanotechnology enhances the specificity of lesions by binding surface-modifying ligands like antibodies, peptides, folic acid to receptors on the surface of inflammatory cells and enables precise localization of colonic inflammation sites.^[36]^ Adhesion molecules or immune cell surface receptors such as MAdCAM-1, macrophage CD44, and TNF-α receptors overexpressed in IBD are common targets.^[13,37]^ For example, integrin α4β7 is a cell surface receptor that is predominantly expressed on memory T lymphocytes. It directs lymphocytes to migrate across the endothelial cell space and to the site of intestinal inflammation by binding to the ligand MAdCAM-1 on intestinal mucosal endothelial cells.^[38,39]^ The nanoparticles modified with anti-integrin α4β7 antibody can selectively bind to intestinal lymphocytes and inhibit their migration to the site of inflammation to achieve anti-inflammatory effects. In addition, targeted nanocarriers targeting intestinal M cells can facilitate antigen-presenting cell uptake for vaccine or immunomodulator delivery.^[40]^ The latest research also explores exosomal biomimetic nanoparticles that use their natural targeted delivery of anti-inflammatory microRNAs.^[41,42]^ The challenge lies in target heterogeneity (differences in targets in different IBD subtypes or stages) and off-target effect control.

### 4.4. Regulate redox homeostasis

Intestinal oxidative stress markers (ROS and malondialdehyde) are elevated, while antioxidant levels are decreased in IBD patients.^[43]^ Nanomaterials can quickly and effectively remove ROS from the site of inflammation by delivering antioxidant enzymes or scavenging ROS, thereby reducing the inflammatory response.^[19,44]^ In addition, by modulating redox homeostasis, nanotechnology help repair and protect the integrity of colonic epithelial cells and enhance intestinal barrier function.^[19]^ The study found that quercetin-loaded poly(lactic-co-glycolic acid) nanoparticles enhanced intestinal antioxidant defense through Nrf2 pathway activation, thereby reducing intestinal inflammation.^[45]^ In addition, ROS-responsive nanosystems, such as thioether bond-modified carriers, enable the release of drugs in high-ROS environments, enabling “smart” treatments.^[46,47]^ It is important to note that nanomaterials themselves may induce oxidative stress and their dosage and biocompatibility need to be rigorously evaluated.

### 4.5. Anti-inflammatory and immunomodulatory

Nanocarriers can efficiently deliver anti-inflammatory drugs (glucocorticoids and JAK inhibitors) or biologics, while enhancing efficacy by modulating immune cell function. For example, IL-10-loaded nanoparticles promote regulatory T cell proliferation and inhibit Th1/Th17 overactivation.^[48,49]^ Macrophage polarization regulation is another focus. Nanoparticles loaded with STAT6 inhibitors can induce polarization of M2-type macrophages and alleviate inflammation.^[50]^ In addition, nanomaterials themselves may have immunomodulatory properties, such as gold nanoparticles that reduce the release of pro-inflammatory factors by inhibiting the NF-κB pathway.^[51,52]^ However, it is important to pay attention to the complex interaction of nanocarriers with the immune system and the risk of complement activation.

### 4.6. Regulate intestinal microbiota

Significant progress has been made in the treatment of IBD by regulating intestinal microbiota homeostasis. The imbalance of intestinal flora is one of the important causes of IBD, which is mainly manifested by excessive proliferation of pathogenic bacteria, decrease of probiotics such as bifidobacteria and lactobacilli, and decreased level of metabolites in microbiota.^[53]^ Nanotechnology provides an innovative strategy for restoring the balance of intestinal microecology through multiple mechanisms such as targeted delivery of antimicrobial drugs, protection of probiotics, regulation of microbiota metabolism, and repair of intestinal barrier.^[54]^

#### 4.6.1. Targeted removal of pathogenic bacteria

Nanocarriers target the surface-specific receptors of pathogenic bacteria through surface modification and release antibiotics or antimicrobial agents to accurately kill pathogenic bacteria and reduce damage to commensal bacteria.^[55]^ Some nanomaterials themselves have antimicrobial activity. Silver nanoparticles inhibit the proliferation of pathogenic bacteria by releasing silver ions to destroy bacterial membrane structure and DNA.^[56]^ And zinc oxide nanoparticles are directly sterilized by producing ROS.^[57]^

#### 4.6.2. Protection and delivery of probiotics

Nano-coating materials, such as sodium alginate and chitosan, are effective in protecting probiotics from damage by gastric acid and digestive enzymes.^[58,59]^ The co-encapsulation of probiotics and prebiotics in nanocarriers can significantly enhance the colonization ability and metabolic activity of microbiota. For example, Bifidobacteria are encapsulated in pH-sensitive hydroxypropyl methylcellulose microcapsules, a design that ensures that Bifidobacteria are released only in a neutral environment in the colon for precise efficacy.^[60]^ Studies have found that liposomes loaded with lactobacillus and inulin can further enhance the colonization effect and metabolic activity of the microflora, providing stronger support for intestinal health.^[61]^

#### 4.6.3. Repair the intestinal mucosal barrier

Nanocarriers deliver microbiota metabolites such as short-chain fatty acids (SCFAs) or modulate their synthesis pathways, which can repair the intestinal barrier and inhibit inflammation. The nanocarriers are loaded with SCFAs such as butyric acid and propionic acid, which are targeted and delivered to colonic epithelial cells, activate G protein-coupled receptors, enhance the expression of tight junction proteins, and repair the intestinal barrier.^[62,63]^ Lipid nanoparticles loaded with butyric acid-CoA transferase gene can promote the synthesis of butyric acid by intestinal microbiota and restore the level of SCFAs.^[62]^ In addition, Nano-coated materials such as hyaluronic acid are used to encapsulate probiotics or beneficial bacteria in the gut to enhance their stability and efficacy in the gut environment. The research team of researcher Liu Jinyao found that this nano-coating can protect *Clostridium butyricum* from gastrointestinal damage and improve its accumulation at the site of intestinal inflammation and injury.^[64]^ The anti-inflammatory properties of hyaluronic acid, combined with the metabolic function of *C butyricum*, are effective in relieving inflammation of the intestinal mucosa and restoring the integrity of the intestinal barrier. Nanocarriers can be used to encapsulate probiotics and prebiotics to enhance the colonization capacity and metabolic activity of the gut microbiota. For example, DNA nanopatch technology has been used to modify the surface of probiotics, significantly improving the probiotics’ tolerance to gastric acid and bile salts.^[65]^ Triggered by low-intensity focused ultrasound, these nanopatches are able to rapidly dissociate and activate probiotic function, thereby significantly reducing inflammation levels and repairing the intestinal barrier in a mouse model of IBD. In addition, Yang research team has developed a novel nanoenzyme SrDy2O4 for the treatment of intestinal diseases. Studies have found that this nanozyme has high gastrointestinal stability, low toxicity and efficient ROS clearance ability, and can target lesion sites through specific in vivo imaging, which can not only reduce inflammation, but also repair the damaged intestinal barrier and restore normal intestinal flora by regulating intestinal immunity, and its therapeutic effect is significantly better than that of traditional drugs.^[66]^

### 4.7. Toxic and side effects of nanotechnology in the treatment of IBD

#### 4.7.1. Material toxicity

Nanoparticles may accumulate in organs such as liver and spleen, causing chronic toxicity or organ damage, especially nondegradable materials, such as metal nanoparticles, which may produce ROS, damage intestinal epithelial cells, and exacerbate inflammation.^[67,68]^ byproducts of biodegradable materials may also alter the intestinal microenvironment or affect microbiota balance.^[15]^

#### 4.7.2. Immune reactions and allergy risk

In the treatment of IBD with nanoparticles, nanoparticles may be recognized by the immune system as a foreign body, triggering an allergic or autoimmune reaction.^[69,70]^ Some nanomaterials or nanoparticles may activate pro-inflammatory signaling pathways or the complement system in the immune system, leading to adverse effects such as increased intestinal inflammation, shortness of breath, increased heart rate, fever, and hypotension.^[71]^

#### 4.7.3. Targeting and nonspecific distribution

When nanopharmaceuticals or nanomaterials are applied in vivo, insufficient targeting may result in the release of the drug in the healthy intestinal region, creating off-target effects, damaging normal tissues, or causing diarrhea.^[72]^ Small particles may enter the bloodstream and affect nontarget organs such as the heart and brain, causing neurological or cardiovascular toxicity.^[73]^

#### 4.7.4. Other side effects

The long-term application of nanotechnology in the treatment of IBD may bring a series of potential risks. On the one hand, the metabolism of nanodrugs may increase the burden on the liver and kidneys, which in turn may lead to abnormal liver and kidney function.^[74]^ In addition, the use of antibiotic-based nanotechnology may induce the emergence of drug-resistant strains, thereby reducing the effectiveness of future treatments. In the production process of nanotechnology, residual chemical reagents may also trigger toxic reactions and pose a threat to patients’ health.

### 4.8. The application value of nanotechnology in the diagnosis of IBD

As a cutting-edge technology, nanotechnology provides a new solution for the diagnosis of IBD. Through surface modification antibodies or aptamers, nanomaterials can specifically capture IBD-related biomarkers such as TNF-α, IL-6, calprotectin, and intestinal microbiota metabolites, significantly improving the detection sensitivity, which is 10 to 100 times higher than that of traditional methods.^[74]^ This highly sensitive test helps in the early diagnosis of IBD, making up for the shortcomings of traditional diagnostic methods. Currently, routine diagnosis of IBD relies on colonoscopy and histopathological analysis, which are invasive and do not allow for early diagnosis.^[75]^ In contrast, nanosensors can detect markers at low concentrations through blood, stool, or urine samples, reducing the need for invasive tests.^[76]^ In addition, oral nanodevices are able to traverse the gastrointestinal tract for real-time detection of local pH, ROS, or inflammatory enzyme activity, and enable noninvasive monitoring via wireless signal transmission.^[16]^ Nanoparticles can also modify targeted molecules to actively aggregate at intestinal inflammation or ulcer sites, enhancing the contrast of MRI, CT, or optical imaging to pinpoint lesion areas.^[77,78]^ This multimodal imaging technology can not only achieve accurate diagnosis, but also initiate targeted therapy at the same time as diagnosis, providing personalized treatment plans for IBD patients.^[79]^ Nanotechnology provides a multidimensional solution from molecular to imaging for the diagnosis of IBD, with ultra-high sensitivity, target specificity, minimally invasive and multimodal integration potential.^[80]^ These advantages make it expected to break through the limitations of traditional methods to achieve early and accurate diagnosis, dynamic monitoring and personalized treatment. With the interdisciplinary development of materials science and biotechnology, the clinical application of nanotechnology in the diagnosis of IBD has broad prospects. Although nanotechnology has shown great potential in the diagnosis of IBD, there are still some challenges. For example, the in vivo safety of nanomaterials, the cost control of large-scale production, the long clinical translation cycle, and the lack of a standardized evaluation system need to be solved urgently. Future research needs to further explore the mechanism of nanomaterials in the diagnosis of IBD, optimize their performance, and verify their efficacy and safety through multicenter clinical trials. In conclusion, nanotechnology brings new hope for the diagnosis of IBD and is expected to become an important clinical tool in the future to improve the diagnosis and treatment outcomes of IBD patients.

### 4.9. Innovation and limitations

This study uses bibliometric methods to systematically sort out the development context of nanotechnology in the field of IBD, and objectively reveals the knowledge structure, distribution of cooperation networks, and the evolution of emerging trends in this field. Through visual analysis, this study identifies the research frontiers and potential development directions that are difficult to capture in traditional narrative reviews, and provides a data-driven empirical basis for the macro background, dynamic evolution and scientific research cooperation model of this field. Although this study strictly follows the bibliometric research methodology, there are still certain limitations. First, in terms of data sources, we only collected literature from the WOSCC database, and only included 2 types of publications, “Article” and “Review,” published in English. This choice may lead us to miss important studies included in other databases (e.g., Scopus, PubMed, Embase, etc) or published in other languages, especially in the interdisciplinary frontier field of nanotechnology and IBD, which may not fully cover all relevant results, thus limiting the breadth of this research perspective to a certain extent. Secondly, in terms of data timeliness, the WOSCC database is continuously updated, and the data captured in this study based on a specific time node (my search time is set to January 1, 2005 to December 31, 2024) is only a cross-section of the dynamic knowledge graph, and the subsequent newly published results may affect the presentation of field hotspots and trends. Future research can further enrich the knowledge picture in this field by expanding data sources and incorporating multi-type literature and multilingual publications.

## 5. Conclusion

This study uses bibliometric methods to systematically analyze the application status and development trend of nanotechnology in the field of IBD research, which provides important methodological supplement and data support for traditional narrative reviews. Unlike subjective literature integration, this study accurately depicts the rapid growth trajectory of this field since 2019 through objective quantitative analysis, identifies the leading positions of the United States, China, and European countries and their cooperative network structures, and reveals the evolution paths of core clusters such as nanodrug delivery systems, targeted therapies, and inflammation regulation. More importantly, based on co-occurrence analysis and reading of key literature, this study prospectively identifies emerging frontiers such as exosomes, macrophage polarization, and gut microbiota regulation, which have not been systematically elaborated in traditional reviews. In addition, through citation gap analysis, this study objectively quantifies the structural bottlenecks in the process of clinical translation of basic research to clinical practice, providing an evidence-based basis for key challenges such as safety evaluation, targeting efficiency improvement, and large-scale production of nanomaterials. In the future, intelligent response nanomaterials, multifunctional nanoplatforms, and personalized nanotechnology will become important development directions. However, the safety, target efficiency, mass production, and clinical translation of nanomaterials are still urgent issues to be addressed. This study provides researchers with a reference to understand the current status, hotspots, and trends in this field, which will help promote the further development of nanotechnology in IBD researches.

## Acknowledgments

We would like to thank Professor Xiaowei Tang from the Department of Gastroenterology, Affiliated Hospital of Southwest Medical University for his guidance. We are grateful for WoSCC database for providing access to the data. Thanks to Citespace, Scimago Graphica, SciExplorer and VOSviewer for free us to perform visual analysis.

## Author contributions

**Data curation:** Lei Liang, Heng Yang, Xiaohe Zhang.

**Formal analysis:** Lei Liang, Heng Yang, Gang Tian.

**Methodology:** Lei Liang, Dexin Wang, Xiubi Zhang, Xiaohe Zhang, Lan Li.

**Project administration:** Weiliang Du.

**Resources:** Lei Liang.

**Software:** Lei Liang, Dexin Wang, Xiubi Zhang, Heng Yang, Xiaohe Zhang.

**Writing – original draft:** Lei Liang, Dexin Wang, Xiubi Zhang, Heng Yang.

**Writing – review & editing:** Lei Liang, Dexin Wang, Xiubi Zhang, Lan Li, Gang Tian, Chaochi Yue, Weiliang Du.

## References

[1] Ng SC Shi HY Hamidi N. Worldwide incidence and prevalence of inflammatory bowel disease in the 21st century: a systematic review of population-based studies. Lancet (London, England). 2017;390:2769–78. 29050646 10.1016/S0140-6736(17)32448-0

[2] Xie A Ji H Liu Z. Modified prebiotic-based “shield” armed probiotics with enhance resistance of gastrointestinal stresses and prolonged intestinal retention for synergistic alleviation of 526 colitis. ACS Nano. 2023;17:14775–91. 37477584 10.1021/acsnano.3c02914

[3] Kaplan GG. The global burden of IBD: from 2015 to 2025. Nat Rev Gastroenterol Hepatol. 2015;12:720–7. 26323879 10.1038/nrgastro.2015.150

[4] Chao L Zhang W Feng Y Gao P Ma J. Pyroptosis: a new insight into intestinal inflammation and cancer. Front Immunol. 2024;15:1117–25. 10.3389/fimmu.2024.1364911 PMC10917886 38455052

[5] Kobayashi T Siegmund B Le Berre C. Ulcerative colitis. Nat Rev Dis Primers. 2020;6:74. 32913180 10.1038/s41572-020-0205-x

[6] Roda G Chien Ng S Kotze PG. Crohn’s disease. Nat Rev Dis Primers. 2020;6:22. 32242028 10.1038/s41572-020-0156-2

[7] Lee Y Sugihara K Gillilland MG Jon S Kamada N Moon JJ. Hyaluronic acid–bilirubin nanomedicine for targeted modulation of dysregulated intestinal barrier, microbiome and immune responses in colitis. Nat Mater. 2019;19:118–26. 31427744 10.1038/s41563-019-0462-9 PMC6923573

[8] Segaert S Hermans C. Clinical signs, pathophysiology and management of cutaneous side effects of anti-tumor necrosis factor agents. Am J Clin Dermatol. 2017;18:771–87. 28597181 10.1007/s40257-017-0296-7

[9] Stallmach A Hagel S Bruns T. Adverse effects of biologics used for treating IBD. Best Pract Res Clin Gastroenterol. 2010;24:167–82. 20227030 10.1016/j.bpg.2010.01.002

[10] Ejeta F. Recent advances of microfluidic platforms for controlled drug delivery in nanomedicine. Drug Des Devel Ther. 2021;15:3881–91. 10.2147/DDDT.S324580 PMC8439440 34531650

[11] Panzarini E Inguscio V Tenuzzo B Carata E Dini L. Nanomaterials and autophagy: new insights in cancer treatment. Cancers. 2013;5:296–319. 24216709 10.3390/cancers5010296 PMC3730308

[12] Xie H He Z Liu Y. Efficient antibacterial agent delivery by mesoporous silica aerogel. ACS Omega. 2022;7:7638–47. 35284760 10.1021/acsomega.1c06198 PMC8908532

[13] Liu P Gao C Chen H. Receptor-mediated targeted drug delivery systems for treatment of inflammatory bowel disease: opportunities and emerging strategies. Acta Pharm. Sin. B. 2021;11:2798–818. 34589398 10.1016/j.apsb.2020.11.003 PMC8463263

[14] Barua S Mitragotri S. Challenges associated with penetration of nanoparticles across cell and tissue barriers: a review of current status and future prospects. Nano Today. 2014;9:223–43. 25132862 10.1016/j.nantod.2014.04.008 PMC4129396

[15] Wang Y Mo Y Sun Y. Intestinal nanoparticle delivery and cellular response: a review of the bidirectional nanoparticle-cell interplay in mucosa based on physiochemical properties. J Nanobiotechnol. 2024;22:669. 10.1186/s12951-024-02930-6 PMC11531169 39487532

[16] Cao L Duan D Peng J. Oral enzyme-responsive nanoprobes for targeted theranostics of inflammatory bowel disease. J Nanobiotechnol. 2024;22:484. 10.1186/s12951-024-02749-1 PMC11321179 39138477

[17] Wu Y Briley K Tao X. Nanoparticle‐based imaging of inflammatory bowel disease. WIREs Nanomed Nanobiotechnol. 2015;8:300–15. 10.1002/wnan.1357 26371464

[18] Cong X Zhang Z Li H Yang YG Zhang Y Sun T. Nanocarriers for targeted drug delivery in the vascular system: focus on endothelium. J Nanobiotechnol. 2024;22:620. 10.1186/s12951-024-02892-9 PMC11470712 39396002

[19] Wang ZH Zeng X Huang W. Bioactive nanomotor enabling efficient intestinal barrier penetration for colorectal cancer therapy. Nat Commun. 2025;16:1678. 39956840 10.1038/s41467-025-57045-9 PMC11830786

[20] Iacucci M Maeda Y Santacroce G Ghosh S. AI-enabled “endo-histo-omics”: breaking down intestinal barriers in IBD. Nat Rev Gastroenterol Hepatol. 2025;22:362–3. 40033062 10.1038/s41575-025-01051-1

[21] Wang Y Xiao Z Wang Z. Multi-omics approaches to decipher the interactions of nanoparticles and biological systems. Nat Rev Bioeng. 2025;3:333–48.

[22] Li Y Liu P Zhang B Chen J Yan Y. Global trends and research hotspots in nanodrug delivery systems for breast cancer therapy: a bibliometric analysis (2013–2023). Discover Oncology. 2025;16:269. 40047951 10.1007/s12672-025-02014-3 PMC11885776

[23] Gu CL Wang ZQ Pan YW Zhu S Gu ZJ. Tungsten-based nanomaterials in the biomedical field: a bibliometric analysis of research progress and prospects. Adv Mater. 2023;35:e2204397. 35906814 10.1002/adma.202204397

[24] Zhang N Li C Chen J Liu X Wang Z Ni J. Research hotspots and frontiers about role of visual perception in stroke: a bibliometric study. Front Neurol. 2022;13:958875. 36188385 10.3389/fneur.2022.958875 PMC9524359

[25] Sun W Gong J Li S. Bibliometric analysis of neuroinflammation and Alzheimer’s disease. Front Aging Neurosci. 2024;16:1423139. 39076205 10.3389/fnagi.2024.1423139 PMC11284157

[26] Xiao B Chen Q Zhang Z. TNFα gene silencing mediated by orally targeted nanoparticles combined with interleukin-22 for synergistic combination therapy of ulcerative colitis. J Controlled Release. 2018;287:235–46. 10.1016/j.jconrel.2018.08.021 PMC6482469 30107214

[27] Xiao B Zhang Z Viennois E. Combination therapy for ulcerative colitis: orally targeted nanoparticles prevent mucosal damage and relieve inflammation. Theranostics. 2016;6:2250–66. 27924161 10.7150/thno.15710 PMC5135446

[28] Zhang M Viennois E Prasad M. Edible ginger-derived nanoparticles: a novel therapeutic approach for the prevention and treatment of inflammatory bowel disease and colitis-associated cancer. Biomaterials. 2016;101:321–40. 27318094 10.1016/j.biomaterials.2016.06.018 PMC4921206

[29] Yufeng Liu YC Zhang H Zhou M. Integrated cascade nanozyme catalyzes in vivo ROS scavenging for anti-inflammatory therapy. Sci Adv. 2020;6:eabb2695. 32832640 10.1126/sciadv.abb2695 PMC7439611

[30] Sriram A Ithape H Singh PK. Deep-insights: nanoengineered gel-based localized drug delivery for arthritis management. Asian J Pharm Sci. 2025;20:101012. 39995751 10.1016/j.ajps.2024.101012 PMC11848107

[31] Agrawal U Sharma R Gupta M Vyas SP. Is nanotechnology a boon for oral drug delivery? Drug Discov Today. 2014;19:1530–46. 24786464 10.1016/j.drudis.2014.04.011

[32] Abaidullah N Muhammad K Waheed Y. Delving into nanoparticle systems for enhanced drug delivery technologies. AAPS PharmSciTech. 2025;26:74. 40038143 10.1208/s12249-025-03063-1

[33] Shi J Zhou J Liu B. Enzyme/ROS dual-sensitive nanoplatform with on-demand Celastrol release capacity for enhanced ulcerative colitis therapy by ROS scavenging, microbiota rebalancing, inflammation alleviating. J Nanobiotechnol. 2024;22:437. 10.1186/s12951-024-02725-9 PMC11282782 39061092

[34] Long J Liang X Ao Z. Stimulus-responsive drug delivery nanoplatforms for inflammatory bowel disease therapy. Acta Biomater. 2024;188:27–47. 39265673 10.1016/j.actbio.2024.09.007

[35] Uzokboev S Akhmadbekov K Nuritdinova R Tawfik SM Lee Y-I. Unveiling the potential of alginate-based nanomaterials in sensing technology and smart delivery applications. Beilstein J Nanotechnol. 2024;15:1077–104. 39188756 10.3762/bjnano.15.88 PMC11346306

[36] Abdelkawi A Slim A Zinoune Z Pathak Y. Surface modification of metallic nanoparticles for targeting drugs.

[37] Di Rienzo A Marinelli L Dimmito MP Toto EC Di Stefano A Cacciatore I. Advancements in inflammatory bowel disease management: from traditional treatments to monoclonal antibodies and future drug delivery systems. Pharmaceutics. 2024;16:1185. 39339221 10.3390/pharmaceutics16091185 PMC11435298

[38] Vermeire S Danese S Sandborn WJ. Efficacy and safety of the anti-mucosal addressin cell adhesion molecule-1 antibody ontamalimab in patients with moderate-to-severe ulcerative colitis or Crohn’s disease. J Crohns Colitis. 2024;18:708–19. 38096402 10.1093/ecco-jcc/jjad199 PMC11140626

[39] Zhao R Gu J Zhao H. Expression of integrin α4β1 and α4β7 on B cells correlates with autoimmune responses in Graves’ disease. Int Immunopharmacol. 2024;142(Pt B):113218. 39317053 10.1016/j.intimp.2024.113218

[40] Li H Chen X Rao S. Recent development of micro-nano carriers for oral antineoplastic drug delivery. Materials today. Bio. 2025;30:101445. 10.1016/j.mtbio.2025.101445 PMC11762190 39866789

[41] Hasan A Ardizzone A Giosa D. The therapeutic potential of MicroRNA-21 in the treatment of spinal cord injury. Curr Issues Mol Biol. 2025;47:70. 39996791 10.3390/cimb47020070 PMC11854632

[42] Luo ZQ Zhou B Xiong H. A bibliometric analysis of exosomes therapy in the treatment of osteoarthritis from 2012 to 2022. J Pain Res. 2023;16:2171–88 37397273 10.2147/JPR.S407050 PMC10312350

[43] Dudzińska E Gryzinska M Ognik K Gil-Kulik P Kocki J Jakovljevic V. Oxidative stress and effect of treatment on the oxidation product decomposition processes in IBD. Oxid Med Cell Longevity. 2018;2018:7918261. 10.1155/2018/7918261 PMC6051053 30057685

[44] Lin J Wei Y Gu X. Nanotherapeutics-mediated restoration of pancreatic homeostasis and intestinal barrier for the treatment of severe acute pancreatitis. J Control Release. 2025;377:93–105. 39542256 10.1016/j.jconrel.2024.11.022

[45] Hussein RM Kandeil MA Soliman HM El-Shahawy AAG. Effect of quercetin-loaded poly (lactic-co-glycolic) acid nanoparticles on lipopolysaccharide-induced memory decline, oxidative stress, amyloidogenesis, neurotransmission, and Nrf2/HO-1 expression. Heliyon. 2024;10:e23527. 38169932 10.1016/j.heliyon.2023.e23527 PMC10758873

[46] Linderman SW DeRidder L Sanjurjo L. Enhancing immunotherapy with tumour-responsive nanomaterials. Nat Rev Clin Oncol. 2025;22:262–82. 40050505 10.1038/s41571-025-01000-6

[47] Patel KD Keskin-Erdogan Z Sawadkar P. Oxidative stress modulating nanomaterials and their biochemical roles in nanomedicine. Nanoscale Horiz. 2024;9:1630–82. 39018043 10.1039/d4nh00171k

[48] Liu F Wang X Ren M. A shielded cascade of targeted nanocarriers spanning multiple microenvironmental barriers for inflammatory disease therapy. J Nanobiotechnology. 2024;22:789. 39710698 10.1186/s12951-024-03075-2 PMC11665124

[49] Zahoor I Bala R Wani SN. Potential role of NSAIDs loaded nano-formulations to treat inflammatory diseases. Inflammopharmacology. 2025;33:1189–207. 39953360 10.1007/s10787-025-01644-x

[50] Wang J Gao H Xie Y. *Lycium barbarum* polysaccharide alleviates dextran sodium sulfate-induced inflammatory bowel disease by regulating M1/M2 macrophage polarization via the STAT1 and STAT6 pathways. Front Pharmacol. 2023;14:1044576. 37144216 10.3389/fphar.2023.1044576 PMC10151498

[51] Elmetwalli A El-Sewedy T Hassan MG. Gold nanoparticles mediate suppression of angiogenesis and breast cancer growth via MMP-9/NF-κB/mTOR and PD-L1/PD-1 signaling: integrative in vitro validation and network pharmacology insights. Naunyn Schmiedebergs Arch Pharmacol. 2025;398:7087–105. 39718609 10.1007/s00210-024-03682-8

[52] Georgeous J AlSawaftah N Abuwatfa WH Husseini GA. Review of gold nanoparticles: synthesis, properties, shapes, cellular uptake, targeting, release mechanisms and applications in drug delivery and therapy. Pharmaceutics. 2024;16:1332. 39458661 10.3390/pharmaceutics16101332 PMC11510955

[53] Szajewska H Scott KP de Meij T. Antibiotic-perturbed microbiota and the role of probiotics. Nat Rev Gastroenterol Hepatol. 2025;22:155–72. 39663462 10.1038/s41575-024-01023-x

[54] Li W Zhan M Wen Y. Recent progress of oral functional nanomaterials for intestinal microbiota regulation. Pharmaceutics. 2024;16:921. 39065618 10.3390/pharmaceutics16070921 PMC11280463

[55] Ahmad M Aduru SV Smith RP Zhao Z Lopatkin AJ. The role of bacterial metabolism in antimicrobial resistance. Nat Rev Microbiol. 2025;23:439–54. 39979446 10.1038/s41579-025-01155-0 PMC12173792

[56] Sczesny NF Wiggers HJ Bueno CZ Chevallier P Copes F Mantovani D. From burst to sustained release: the effect of antibiotic structure incorporated into chitosan-based films. Antibiotics (Basel). 2024;13:1055. 39596749 10.3390/antibiotics13111055 PMC11591004

[57] Shanmugam R Govindharaj S Arunkumar P Sai Sanjana G Manigandan P. Preparation of a herbal mouthwash with lemongrass and mint-mediated zinc oxide nanoparticles and evaluation of its antimicrobial and cytotoxic properties. Cureus. 2024;16:e53671. 38455834 10.7759/cureus.53671 PMC10918288

[58] Zhou J Li M Chen Q. Programmable probiotics modulate inflammation and gut microbiota for inflammatory bowel disease treatment after effective oral delivery. Nat Commun. 2022;13:3432. 35701435 10.1038/s41467-022-31171-0 PMC9198027

[59] Yin H Li J Huang H. Microencapsulated phages show prolonged stability in gastrointestinal environments and high therapeutic efficiency to treat *Escherichia coli* O157:H7 infection. Vet Res. 2021;52:118. 34521472 10.1186/s13567-021-00991-1 PMC8439058

[60] Voblikova T Laricheva K Chandra Sekar CM. Bifidobacteria encapsulation and viability of probiotic culture during oral delivery in a milk drink matrix. Int. J. Food Sci. 2023;2023:8484835. 37547341 10.1155/2023/8484835 PMC10400300

[61] Shi F Gao Y Shen M. Preliminary exploration of inulin and inulin liposome on DSS-induced colitis remission. J Drug Delivery Sci Technol. 2023;88:104911.

[62] Nshanian M Gruber JJ Geller BS. Short-chain fatty acid metabolites propionate and butyrate are unique epigenetic regulatory elements linking diet, metabolism and gene expression. Nat Metab. 2025;7:196–211. 39789354 10.1038/s42255-024-01191-9 PMC11774759

[63] Mann ER Lam YK Uhlig HH. Short-chain fatty acids: linking diet, the microbiome and immunity. Nat Rev Immunol. 2024;24:577–95. 38565643 10.1038/s41577-024-01014-8

[64] Deng B Lin S Wang Y. Hyaluronic acid-nanocoated bacteria generate an anti-inflammatory tissue-repair effect in impaired gut and extraintestinal organs. Adv Mater. 2025;37:e2412783. 39568244 10.1002/adma.202412783

[65] Jin X Li H Pan S. DNA nanopatch-specific modification of probiotics for ultrasound-triggered inflammatory bowel disease therapy. J Am Chem Soc. 2024;146:33817–31. 39508560 10.1021/jacs.4c12139

[66] Zhao X Yu Y Xu X. Machine learning-assisted high-throughput screening of nanozymes for ulcerative colitis. Adv Mater. 2025;37:e2417536. 39801185 10.1002/adma.202417536

[67] Raj CTD Muthukumar K Dahms HU James RA Kandaswamy S. Structural characterization, antioxidant and anti-uropathogenic potential of biogenic silver nanoparticles using brown seaweed *Turbinaria ornata*. Front Microbiol. 2023;14:1072043. 37727290 10.3389/fmicb.2023.1072043 PMC10505674

[68] De I Singh R Kumar S. Short term biodistribution and in vivo toxicity assessment of intravenously injected pristine graphene oxide nanoflakes in SD rats. Toxicol Res (Camb). 2024;13:tfae058. 38617714 10.1093/toxres/tfae058 PMC11014786

[69] Nguyen VP Qian W Zhe J. Renally clearable ultraminiature chain-like gold nanoparticle clusters for multimodal molecular imaging of choroidal neovascularization. Adv Mater. 2023;35:e2302069. 37285214 10.1002/adma.202302069 PMC10509731

[70] Mancuso C Barisani D. Food additives can act as triggering factors in celiac disease: current knowledge based on a critical review of the literature. World J Clin Cases. 2019;7:917–27. 31119137 10.12998/wjcc.v7.i8.917 PMC6509268

[71] Li Y Jacques S Gaikwad H. Inhibition of acute complement responses towards bolus-injected nanoparticles using targeted short-circulating regulatory proteins. Nat Nanotechnol. 2024;19:246–54. 37798566 10.1038/s41565-023-01514-z PMC11034866

[72] Cho Y Seo EU Hwang KS Kim H Choi J Kim HN. Evaluation of size-dependent uptake, transport and cytotoxicity of polystyrene microplastic in a blood-brain barrier (BBB) model. Nano Converg. 2024;11:40. 39406944 10.1186/s40580-024-00448-z PMC11480280

[73] Cheng Y Chen Z Yang S. Nanomaterials-induced toxicity on cardiac myocytes and tissues, and emerging toxicity assessment techniques. Sci Total Environ. 2021;800:149584. 34399324 10.1016/j.scitotenv.2021.149584

[74] Jiang HY Shao B Wang HD. Analysis of nanomedicine applications for inflammatory bowel disease: structural and temporal dynamics, research hotspots, and emerging trends. Front Pharmacol. 2025;15:3073–82. 10.3389/fphar.2024.1523052 PMC11750799 39845796

[75] Fu W Xu L Chen Z. Recent advances on emerging nanomaterials for diagnosis and treatment of inflammatory bowel disease. J Control Release. 2023;363:149–79. 37741461 10.1016/j.jconrel.2023.09.033

[76] Zhou D Yin Y Zhu Z. Orally administered platinum nanomarkers for urinary monitoring of inflammatory bowel disease. ACS Nano. 2022;16:18503–14. 36300570 10.1021/acsnano.2c06705

[77] Yue NN Xu HM Xu J. Application of nanoparticles in the diagnosis of gastrointestinal diseases: a complete future perspective. Int J Nanomed. 2023;18:4143–70. 10.2147/IJN.S413141 PMC10387254 37525691

[78] Luo Y Gao C Chen W Zhou K Xu M. Molecular magnetic resonance imaging with contrast agents for assessment of inflammatory bowel disease: a systematic review. Contrast Media & Molecular Imaging. 2020;2020:4764985. 32454803 10.1155/2020/4764985 PMC7225866

[79] Maksymova L Pilger YA Nuhn L Van Ginderachter JA. Nanobodies targeting the tumor microenvironment and their formulation as nanomedicines. Mol Cancer. 2025;24:65. 40033293 10.1186/s12943-025-02270-5 PMC11877942

[80] Arvejeh PM. Nanobiomaterials & nanomedicine. J Transl Med. 2024;22:1154. 39731150 10.1186/s12967-024-06005-w PMC11681672

